# Incidence, prevalence and risk factors for hepatitis C in Danish prisons

**DOI:** 10.1371/journal.pone.0220297

**Published:** 2019-07-26

**Authors:** Jacob Søholm, Dorte Kinggaard Holm, Belinda Mössner, Lone Wulff Madsen, Janne Fuglsang Hansen, Nina Weis, Agnes Pernille Sauer, Tahany Awad, Peer Brehm Christensen

**Affiliations:** 1 Department of Infectious Diseases, Odense University Hospital, Odense, Denmark; 2 OPEN, Odense Patient data Explorative Network, Odense University Hospital, Odense, Denmark; 3 Clinical Institute, University of Southern Denmark, Odense, Denmark; 4 Department of Clinical Immunology, Odense University Hospital, Odense, Denmark; 5 Department of Infectious Diseases, Copenhagen University Hospital, Hvidovre, Denmark; 6 Department of Clinical Medicine, Faculty of Health and Medical Sciences, University of Copenhagen, Copenhagen, Denmark; 7 Department of Infectious Diseases, Aarhus University Hospital, Aarhus, Denmark; 8 Medical Affairs, AbbVie A/S, Copenhagen, Denmark; Hofstra University, UNITED STATES

## Abstract

Hepatitis C virus (HCV) infection is prevalent among people in prison and prisons could therefore represent a unique opportunity to test risk groups for HCV. The aim of this sero-epidemiological study was to determine the incidence and prevalence of HCV infection and the corresponding risk factors in Danish prisons. Participants, recruited from eight Danish prisons, were tested for HCV using dried blood spots and filled out a questionaire with demographic data and risk factors for HCV infection. In total, 76.9% (801/1041) of all eligible prisoners consented to participate. The prevalence of HCV RNA positive prisoners was 4.2% (34/801) and the in-prison incidence rate was 0.7–1.0 per 100PY overall and 18-24/100PY among PWIDs. Infected prisoners were older than the overall population with a mean age of 42 years and only 17.6% (6/34) were younger than 35 years. The prevalence of PWID was 8.5% (68/801) and only 3% (2/68) of PWID were younger than 25 years. Among the PWID, 85.3% (58/68) had ever received opioid substitution therapy (OST) and 47.1% (32/68) were currently receiving OST. Risk factors associated with HCV infection were intravenous drug use, age ≥ 40 years, and being incarcerated ≥ 10 years. In conclusion, the prevalence of PWID in Danish prisons is low, possibly reflecting a decrease in injecting among the younger generation. This together with OST coverage could explain the low prevalence of HCV infection. However among PWIDs in prison the incidence remains high, suggesting a need for improved HCV prevention in prison.

## Introduction

Chronic hepatitis C (CHC) is a major cause of chronic liver disease worldwide with an estimated 71 million people infected and a 20-year risk for developing cirrhosis of up to 30% [1] leading to 400,000 deaths annually from hepatocellular carcinoma (HCC) and end-stage liver disease [1, 2]. Well tolerated therapies for CHC with cure rates above 95% are now available [3]. Treatment leading to sustained virological response (SVR) is associated with decrease in liver stiffness [4, 5] and a reduction in the risk of progressing to cirrhosis, HCC and end-stage liver disease [6, 7].

WHO has set an ambitious goal of eliminating viral hepatitis as a global health threat by 2030. This includes reducing new hepatitis C virus (HCV) infections by 90% and deaths by 65% [8].

However, treatment of those infected with HCV is currently limited by the fact that only a fraction of those infected have been diagnosed. In Denmark, the estimated prevalence of CHC is below 0.5% and only half of the estimated CHC population has been diagnosed, emphasizing the need for further screening [9, 10].

For screening to be cost effective in countries with low prevalence it should be targeted at high risk populations [11, 12] who should be tested repeatedly in a “treatment as prevention” strategy to reach the WHO goals [13].

Prisoners have a higher prevalence of CHC than the population as a whole, mainly because of the incarceration of people who inject drugs (PWID) [14–16] who are the main risk group for HCV. In the European Union country based anti-HCV prevalence among PWID ranges from 14.6% to 84.3%, and in Denmark this prevalence is estimated to 40% [9, 17].

In a recent meta-analysis Dolan et al. estimated the average prevalence of antibodies against HCV (anti-HCV) in prisoners in Western Europe to be 15.5% [14]. Prisons could therefore represent a unique opportunity to test risk groups for HCV, but the uptake is generally low [16, 18, 19] and the prevalence of HCV among prisoners vary widely in individual studies, ranging from 1.1% to > 80% [14, 17]. The only available data on HCV infection among Danish prisoners comes from a study by Christensen et al from 1997 [20] e that included 325 prisoners, 140 (43.1%) of which were PWID, in a high security prison on Funen, Denmark. The prevalence of HCV infection was 28.9% overall and 56.4% among PWID and the incidence rate was 2.7/100 PY in the whole population and 25.2/100 PY among PWID.

Identifying risk factors for HCV infection in prisoners could help identify risk groups for whom getting tested should be a priority. In a meta-analysis of risk factors for HCV infection among prisoners involving 30 studies and over 30.000 prisoners injecting drug use, getting a tattoo and female gender were found to be associated with being infected with HCV [21].

The aim of this study was to determine the prevalence and incidence of HCV infection and the corresponding risk factors for HCV infection in Danish prisons. We furthermore extracted register data and cross referenced with self-report to identify previously diagnosed infections.

## Material and methods

### Study area and population

The study was conducted in one low security prison, five high security prisons and two prisons with both high security and low security wards, in two of the five Regions in Denmark: in the Southern and Midwestern part of Denmark, between September 2016 and August 2017. The prisons had a capacity of 1,280 inmates and approximately 3,000 admissions per year, which represented approximately one-third (1,280/3,590) of the Danish prison population at the time. Foreign prisoners made up 10% of all prisoners in Denmark in 2016 and 2017 and the average occupancy was 95%. Prisoners in Danish prisons have access to opioid substitution therapy (OST) but do not have access to needle exchange. All prisoners are offered a consultation with a prison nurse on admission and can be tested for blood borne viruses, if the prisoner reports or is known to have risk behavior, but there is no formalized screening for HCV or other viruses in Danish prisons.

The included prisons were chosen based on the presence of outpatient clinics in the high security prisons in the two regions, served by the main department of infectious diseases in each region, in order to ensure optimal linkage to care of prisoners diagnosed with HCV infection. The prisons included in the study received prisoners from the whole country and were representative of the prisons in Denmark.

All prisoners aged ≥18 years with a Danish unique, ten-digit Personal Identification Number (PIN), who were present in prison on the survey days, were offered participation. Foreign prisoners who did not have a Danish PIN, and were to be expelled from the country at release from prison, were on request from prison authorities excluded from the study, as they could not be referred for treatment if diagnosed with CHC.

One week before the study visit, prisoners were informed of the study by posters. On the day of the study visit, the prisoners did not go to work but remained in their wing, without loss of payment for the day’s work. All prisoners were given a plenary introduction to the study before being invited to participate. Prisoners were identified by their PIN. Informed consent was obtained in writing from all participants. Participants were able to opt out of the study at any time after inclusion without it affecting subsequent treatment or their sentence. The study was conducted by the authors independently of the prison authorities, and the study team was not part of prison staff. Participants with a positive test for HCV were informed about their diagnosis and referred for further assessment and treatment by the first author.

Ethical approval for the study was given by the Regional Committees on Health Research Ethics for Southern Denmark. The study was accepted by the prisoners’ spokesmen and participants received no monetary incentive.

Demographic and behavioral data were collected in private through an individual, structured interview conducted by a medical doctor using a questionnaire developed for the study (see appendix). We defined all illicit drugs except cannabis as hard drugs and People Who Injected Drugs ever as PWIDs.

### Laboratory methods

Dried blood spot (DBS) samples were collected from all consenting participants after completing the interview as previously described [22]. DBS were not used routinely in prisons in Denmark during the study period. The DBS were obtained on Whatman 903 protein saver card (Sigma-Aldrich, Copenhagen, Denmark) and were allowed to dry for 1 to 3 days before they were eluted and tested for anti-HCV using the Architect System (Abbott Diagnostics, Delkenheim. Germany). Serology positive samples were tested for the presence of HCV RNA by Nucleic Acid Amplification Testing using multiple primers and the Procleix Panther System (Grifols Diagnostic Solutions, Allschill, Switzerland). Remaining blood spot samples on the Whatman card were stored at -80°C.

To test for incidence, all samples were tested for the presence of HCV RNA in pools of five and samples in positive pools were tested individually. The DBS samples were thawed and eluted by incubating with 1,150 μL SPER buffer (Roche Diagnostics, Basel, Switzerland) for 10 min at 56°C on a thermomixer (shaking at 1,000 rpm) and the testing was performed using the cobas MPX assay at the cobas6800 system (Roche Diagnostics, Basel, Switzerland).

### HCV-incidence calculation

Participants who were HCV RNA-positive and anti-HCV-negative were assumed to be newly infected with HCV. The number of these participants relative to the anti-HCV negative participants was used to estimate incidence using the following calculation: I = [(365/T)n]/[(N-n) + (365/T)n] where I = incidence, T = estimated mean duration of the anti-HCV-negative/ HCV RNA-positive window period, n = number of incident infections (anti-HCV-negative HCV RNA-positive) and N = number of susceptible (anti-HCV-negatives, both HCV RNA-negative and positive).

The window period from positive HCV RNA to detection of anti-HCV was assumed to be between 51 to 75 days [23, 24].

Only prisoners, who had been incarcerated for at least 75 days in high security prisons, were included in the analysis, in order to exclude transmissions prior to incarceration.

### Register data

We used data from the research laboratory database (DANVIR) that included all patients tested for HCV and HBV in 14 of the 18 Danish laboratories performing HCV and HBV tests (excluding blood donors). The laboratories contributing to the database served an estimated 85% of the Danish population and included data on 177,453 persons tested for hepatitis C. Data were included from the initiation of electronically stored test results used by all laboratories from 2000 onwards. The data from this database were cross referenced with the participant list using their unique civil registration number to identify positive tests among the participants.

As the database has been incompletely updated from 2013 and onwards the data did not allow identification of infections hereafter missed by DBS.

The following national source registers were used to search for a previous diagnosis of viral hepatitis C:

*Hospital Register:* All discharge diagnoses for inpatients from 1977 and all hospital outpatient visits for patients treated in Danish hospitals. We extracted all individuals who were registered with chronic hepatitis C (ICD-10 diagnosis 18.2).

*The Danish Database for hepatitis B and C (DANHEP):* A national clinical database containing all patients ≥ 15 years of age with chronic viral hepatitis B or C, who have attended specialist care in Denmark since 2002, regardless of the year of first contact.

*Communicable Disease Register:* This national public register of notifiable diseases contains mandatory report forms from diagnosing physicians of acute and chronic viral hepatitis B and C.

### Statistical analysis

Non parametric tests were used for univariate analysis of baseline data (chi-square test, Fischer’s exact test or Mann-Whitney test as appropriate).

Univariate and multivariate logistic regression was performed to identify factors associated with past or present infection with HCV. The multivariate model was checked for significant interactions and the number of variables was reduced using backwards elimination and likelihood ratio tests to determine the best model.

Level of significance was set to *p* < 0.05. Data processing and statistical analysis was performed using STATA 15 IC software (Statacorp LP, College Station, TX).

## Results

In all 76.9% (801/1041) of all eligible prisoners consented to participate in the study. Data were available for 70.8% (182/257) of the non-participant who did not differ by gender or proportion tested registered in the national registers, but the median age of non-participants was 38 years (IQR 27–46) compared to 30 years (IQR 24–39) among participants.

Baseline characteristics of the overall population and of the participants with current or past HCV infection are described in Table 1.

**Table 1:** Main characteristics according to the presence of current or past HCV infection in prisoners in the Southern and Middle Regions of Denmark, 2016–2017.

	Never HCV infection (*n* = 740)	Ever HCV infection (*n* = 61)	p	Total (*n* = 801)
Sex, n (%)			0.429	
Female	22 (3.0)	3 (4.9)		25 (3.1)
Male	718 (97.0)	58 (95.1)		776 (96.9)
Age in years (median, [IQR])	29 [24–38]	42 [37–47]	**<0.001**	30 [24–39]
Country of birth, n (%)			**0.035**	
Not Denmark	186 (25.1)	8 (13.1)		194 (24.2)
Denmark	554 (74.9)	53 (86.9)		607 (75.8)
Lifetime years in prison (median [IQR])	4 [2–7]	10 [6–16]	**<0.001**	4 [2–7]
Ever smoked cannabis, n (%)	521 (70.6)	56 (91.8)	**<0.001**	577 (72.2)
Grams/week cannabis (median [IQR])	15 [10–35]	15 [14–35]	0.861	15 [10–35]
Years of smoking cannabis (median [IQR])	8 [4–13]	20 [12–30]	**<0.001**	9 [5–15]
Ever injected anabolic steroids, n (%)	284 (38.4)	18 (29.5)	0.169	302 (37.7)
Ever injected anabolic steroids in prison, n (%)	46 (6.2)	1 (1.6)	0.249	47 (5.9)
Ever used heroin, n (%)	92 (12.4)	55 (90.2)	**<0.001**	147 (18.4)
Ever used cocaine, n (%)	566 (76.5)	52 (82.3)	0.117	618 (77.2)
Ever used amphetamine, n (%)	342 (46.2)	46 (75.4)	**<0.001**	388 (48.4)
Ever shared snorting straw, n (%)	541 (73.1)	43 (70.5)	0.659	584 (72.9)
Ever shared snorting straw in prison, n (%)	183 (24.7)	24 (39.3)	**0.012**	207 (25.8)
Ever injected drugs, n (%)	17 (2.3)	51 (83.6)	**<0.001**	68 (8.5)
Ever injected drugs in prison, n (%)	4 (0.5)	25 (41.0)	**<0.001**	29 (3.6)
Ever shared drug paraphernaliaᵃ, n (%)	6 (0.8)	37 (60.7)	**<0.001**	43 (5.4)
Ever shared drug paraphernaliaᵃ in prison, n (%)	2 (0.3)	13 (21.3)	**<0.001**	15 (1.9)
Ever opioid agonist therapy, n (%)	45 (6.1)	50 (82.0)	**<0.001**	95 (11.7)
Current opioid antagonist therapy, n (%)	24 (3.2)	26 (42.6)	**<0.001**	50 (6.2)
Tattoo, n (%)	486 (65.7)	48 (78.7)	**0.038**	534 (66.7)
Prison Tattoo, n (%)	68 (9.2)	15 (24.6)	**<0.001**	83 (10.4)
Sexual preference, n (%)			0.153	
Heterosexual	721 (97.4)	58 (95.1)		779 (97.3)
Bi- or homosexual	15 (2.0)	3 (4.9)		18 (2.2)
Ever had a sexual transmitted disease, n (%)	291 (39.3)	18 (29.5)	0.122	309 (38.5)

Abbreviations: *HCV* Hepatitis C Virus, *IQR* Interquartile Range

ᵃNeedles, spoons, water or filters

### HCV prevalence

**Fig 1:**
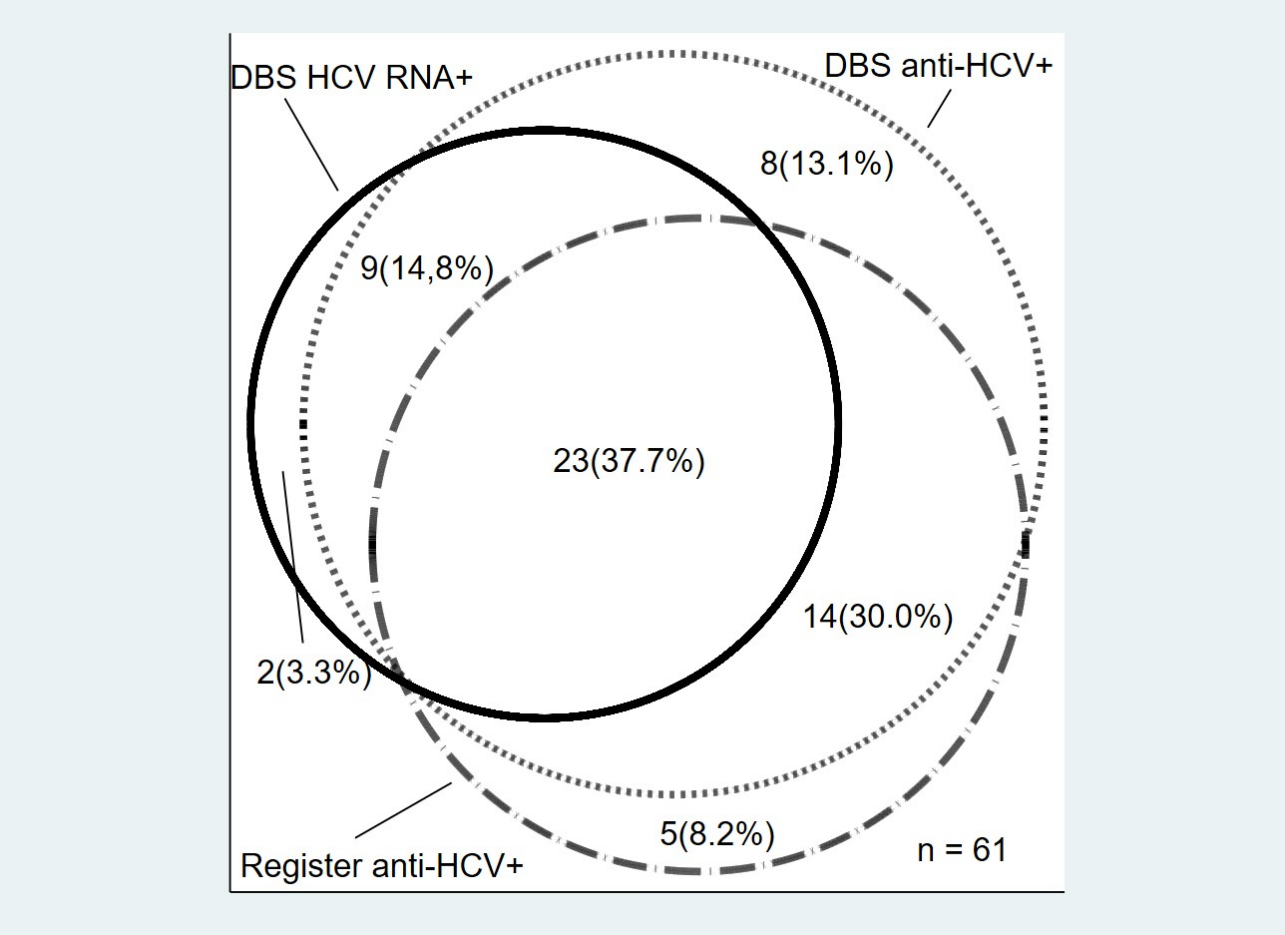
Serological markers for HCV infection from dried blood spot and registers. **Venn diagram showing overlap of individual markers with figures in actual size.** Abbreviations: *HCV* Hepatitis C Virus, *DBS* dried blood spot.

The overall prevalence of anti-HCV positive participants was 7.4% (59/801, 95% CI: 5.7%-9.4%) and of these 92% (54/59, 95% CI: 81.3–97.2) had a positive test for anti-HCV on DBS (Fig 1). The five prisoners who only had a positive test for anti-HCV in the registers did not differ from those with a positive anti-HCV on DBS in terms of age, sex, intravenous drug use or time spent in prison.

Current HCV infection with a positive HCV RNA on DBS was found in 4.2% (34/801, 95% CI: 3.0%-5.9%) while a positive HCV RNA was found in 39.7%, (27/68, 95% CI: 28.0–52.3%) of PWID compared to 1.0% (7/733, 95% CI: 0.4–2.0%) in those who did not report intravenous drug use (p < 0.001).

The rate of active infection among those with a positive anti-HCV was 54.2% (32/59, 95% CI: 40.8%-67.3%) and in those with past HCV infection previous treatment for HCV was reported in 18.5% (5/27, 95% CI: 6.3%-38.1%).

**Fig 2:**
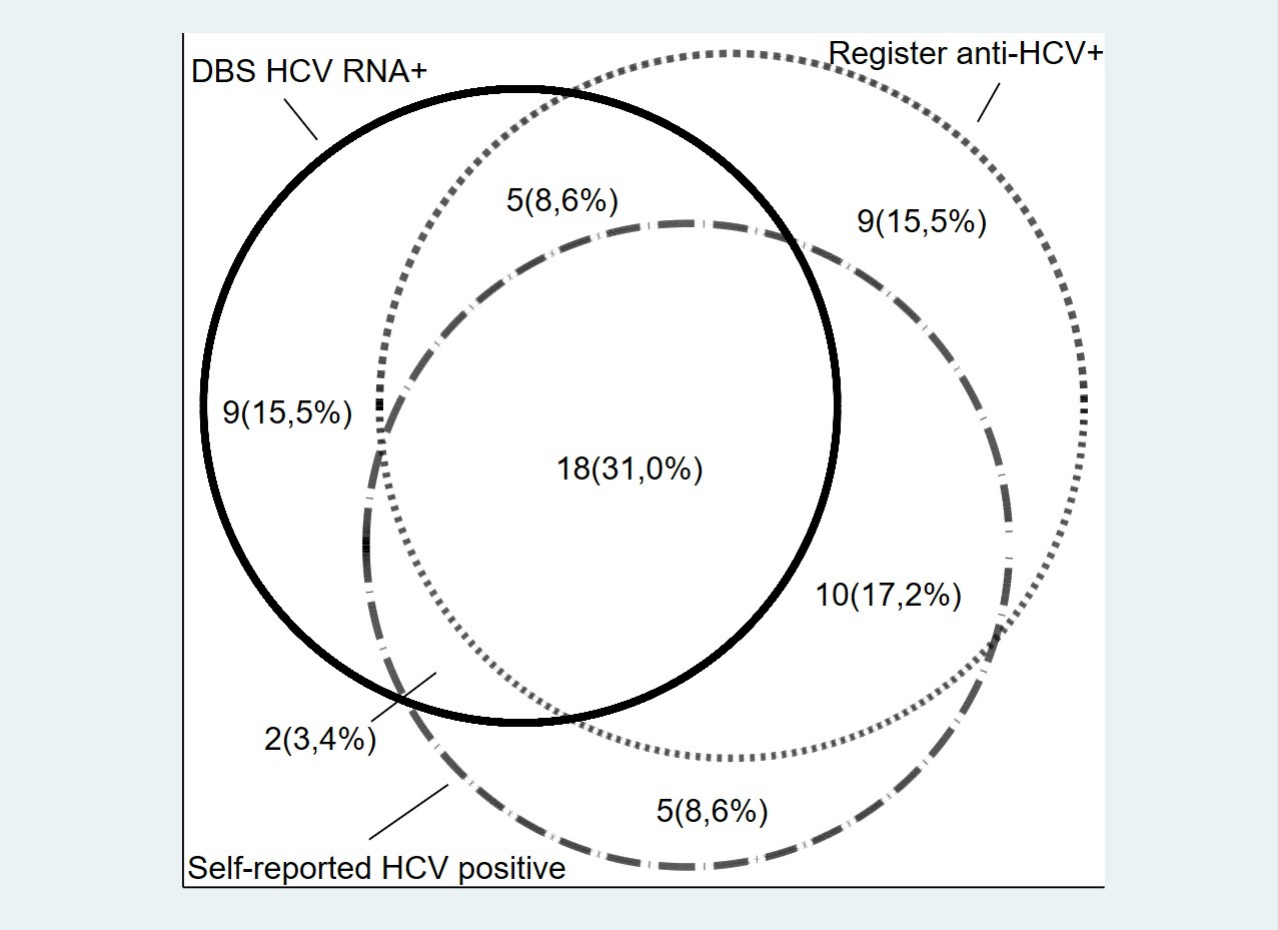
Self-reporting of HCV infection and previous HCV serology in HCV RNA positive prisoners. **Venn diagram showing overlap with figures in actual size.** Abbreviations: *HCV* Hepatitis C Virus, *DBS* dried blood spot.

A total of 29.3% (235/801, 95% CI: 26.2%-32.6%) reported having been tested for hepatitis C previously and 4.4% (35/801, 95% CI: 3.1%-6.0%) reported being infected with hepatitis C of which 57.1% (20/35, 95% CI: 39.4%-73.7%) were currently infected and 28.6% (10/35, 95% CI: 14.6%-46.3%) had a previous positive anti-HCV test in the registers (Fig 2).

Combining self-reporting with register data of a positive anti-HCV test identified 73.5% (25/34, 95% CI: 55.6%-87.1%) of those with current infection (Fig 2). The 26.5% (9/34, 95% CI: 12.9%-44.4%) with a previously undiagnosed infection did not differ from those with a known infection in terms of age, sex or intravenous drug use, but had spent less time in prison during their lifetime. Of those reporting to be infected with HCV 70% (14/20) reported to be attending care for their infection. Among prisoners self-reporting active HCV infection a sensitivity and specificity of respectively 62.5% and 93.6% was found and, assuming a prevalence of HCV infection of 4.2%, the positive predictive value (PPV) and negative predictive value (NPV) of self-reported infection was 30% and 98%.

### HCV incidence

The crude observed incidence in the cohort was 1.4–2.0/100personyears (PY). Both of the two participants who were HCV RNA positive and anti-HCV negative were registered as having a negative anti-HCV test in the registers. Only one prisoner had been incarcerated >75 days in a maximum security prison at the time of the test resulting in an in-prison incidence rates of 0.7–1.0 per 100PY assuming a window period of 51–75 days. The included prisoner reported injection drug use and having injected drugs in prison previously. The incidence rate among the 23 antibody-negative PWID was 18.1–24.5/100 PY. Of these 23, seven reported ever injecting in prison and 2 reported injecting during the present incarceration.

### Drug use among prisoners

Of all participants 81.5% (653/801, 95% CI: 78.7%-84.2%) had used some kind of illicit drug not including hashish (Table 1).

Among PWID 95.6%, (65/68, 95% CI: 87.6–99.1%) reported injecting heroin while the last three reported injecting either amphetamine or cocaine.

The median age of PWID was 41 years (IQR 33–46 years, range 22–59 years) and the median duration of injecting drug use was 10 years (IQR 3–18 years). A majority of 62.7% (42/67, 95% CI: 50.0%-74.2%) had stopped injecting before the year they were incarcerated while 9.0% (6/67, 95% CI: 3.3%-18.5%) had injected during the current incarceration, four of which reported injecting within the last year.

Among PWID 85.3% (58/68, 95% CI: 74.6%-92.7%) had ever received opioid substitution therapy (OST) and 47.1% (32/68, 95% CI: 34.8%-59.6%) were currently on OST

### Risk factors

As there was no statistically significant difference between participants with past or present HCV infection in any of the chosen risk factors in Table 2 both groups were included in the risk factor analysis. Several risk factors were significantly associated with having been exposed to HCV in the univariate analysis, including age ≥ 40 years, lifetime imprisonment ≥ 10 year, injecting drug use, injecting drugs in prison, getting prison tattoos and being born in Denmark. In the multivariate analysis only injecting drug use, age ≥ 40 years and a lifetime of imprisonment ≥ 10 years were associated with having been exposed to HCV.

**Table 2:** Factors associated with HCV infection in prisoners from the southern and middle regions of Denmark, (n = 801), 2016–2017.

	Univariate analysis			Multivariate analysis	
	n/N	OR (95% CI)	p	OR (95% CI)	p
Age			<0.001		0.028
< 40 years	22/609	1		1	
≥ 40 years	39/192	6.8 (3.9–11.8)		2.9 (1.1–7.4)	
Country of birth			0.04		
Not Denmark	8/194	1			
Denmark	53/607	2.2 (1.0–4.8)			
Lifetime years in prison			<0.001		<0.001
< 10 years	24/668	1		1	
≥ 10 years	37/133	10.3 (5.9–18.0)		6.0 (2.2–16.5)	
Shared snorting straw in prison			0.014		
No	37/594	1			
Yes	24/207	2.0 (1.15–3.4)			
Injected drug use			<0.001		<0.001
No	10/733	1		1	
Yes	51/68	217 (94.5–498)		182 (71.6–465)	
Injecting drug use in prison			<0.001		
No	36/772	1			
Yes	25/29	128 (42.2–387)			
Prison tattoo			<0.001		
No	46/718	1			
Yes	15/83	3.2 (1.7–6.1)			

n/N: number of prisoners with past or present HCV infection/total number of prisoners for a specific category

Abbreviations: *HCV* Hepatitis C Virus, *OR* Odds Ratio, *CI* Confidence Interval

Among the seven HCV positive participants without reported intravenous drug use, all reported having used hard drugs and of these, three reported previous heroin use. Three had shared a snorting straw while in prison and two had prison tattoos. All seven were heterosexual and none reported having had a sexually transmitted disease.

## Discussion

This is the first study to be conducted on the prevalence and incidence of hepatitis C among Danish prisoners in 20 years. In our study which included approximately one-third of the Danish prison population we found an overall prevalence of active HCV infection of 4.2% (34/801) and an incidence of 0.7–1.0/100PY. However the estimated incidence among PWIDs in prison (18-24/100PY) remained very high and comparable to our 20 years old findings. Almost three-quarter (25/34) of the infected prisoners had been diagnosed with hepatitis C previously when combining information from self-report and register data, but less than half (14/34) were attending care. Infected prisoners were older than uninfected, with a mean age of 42 years compared to 30 years in uninfected prisoners. Only 17.6% of the HCV infected prisoners were younger than 35 years of age and none were younger than 25 years.

Correspondingly, the prevalence of prisoners who ever injected drugs was 8.5% (68/801). The mean age in this group was 41 years and only one prisoner younger than 25 years had injected heroin. Among the PWID 85.3% had ever received OST.

The only risk factors found to be associated with HCV infection in the multivariate regression analysis were injecting drug use with an increasing OR with longer duration of injection, older age and longer total time imprisonedlonger total time imprisoned.

A high participation rate of 76.9% was achieved in this study, which is in the upper range of the 40% - 80% that is generally achieved in hepatitis C serosurveys and routine testing in prisons [18]. A risk of selection bias cannot be excluded, as participation could be related to the prisoner’s risk of being infected. Prisoners who were aware of being infected or suspected it because of previous or present intravenous drug use could have declined participation as they considered it redundant or they could fear stigmatization. Conversely, prisoners who never injected could have declined participation, because they did not consider themselves at risk of being infected.

One of this study’s strengths was that information about age, gender and previous diagnosis of HCV infection were available on the majority of non-participant, which is not available in most studies and thus could estimate the selection bias in the study. Non-participants were older than participants which could indicate a higher prevalence of PWID and HCV infection among the non-participants as PWID and participants with HCV infection were older than the other participants, but there was no difference between participants and non-participants prevalence of HCV in the used databases, indicating that there was no selection bias in the study with regards to previous test and diagnosis.

Furthermore, according to the Danish Prison and Probation Service 16.6% of all prisoners in Denmark in 2016 reported having used opioids [25] which is comparable to the 18.4% who reported using heroin in our study indicating that we did not have an over- or underrepresentation of heroin users.

A weakness of the study was the use of DBS, as it was not possible to confirm positive results by a second venous sampling. HCV-RNA positive inmates were referred for clinical evaluation and were all confirmed, but it is possible that we may have overlooked some weakly positive chronic infections as the sensitivity of the assay was 100 IU/mL for HCV-RNA [22]. The discrepancy between historical registers data/self-reported infection and a new test was to be expected and we found no indication that we misclassified a significant proportion of the tested.

Another weakness of our study was that it only included prisons from two of the five regions in Denmark as the demographic characteristics of prisoners in other regions of Denmark could have differed from those found in our study. This could be especially true in the Region of Copenhagen, where a higher proportion of those diagnosed with HCV resides, compared to other areas of Denmark [9].

The prevalence of anti-HCV positivity of 7.6% in this study is lower than the overall 15.5% anti-HCV positivity among Western European prison inmates found by Dolan et al[14], but a lower prevalence is generally seen in studies from Northern European countries and in more recent studies[14, 26–28]. This suggests a geographical trend that has also been found in the general population as well as in PWID [29]. In a study looking at HCV in prisoners and the general population in Southern France over time, Roux et al. found a declining prevalence of 7.9% - 3.5% from 2004–2010 [28].

The marked decline in HCV prevalence in Danish prisoners from 28.9% to 4.2% over a 20 year period was driven by a decline in the prevalence of PWID in Danish prisons in the same period from 43.1% to 8.5% which seems to mirror a trend in the general population in Denmark. The latest estimate of the PWID population in Denmark dates back to 2009, where it was found to be approximately 13,000 corresponding to 3.6/1,000 (95% CI: 2.8–4.6) among the 15–64 years old [30], but other data support a recent decline in injection drug use among drug abusers. There has been a marked decline in those treated for drug addiction in Denmark who reports heroin as their main drug. In 2003 15.3% of the 18–24 year old and 30.2% across all age groups of those treated for drug addiction in Denmark reported using opioids as their main drug whereas in 2015 it had dropped to 1.6% and 5.9% respectively [30]. Among those treated for heroin addiction in 2015 44% were enrolled for the first time and of these only 10% reported injecting in contrast to 47% among those who had also been treated previously [30].

That fewer young people in Denmark use heroin and start injecting drugs leading to an ageing and diminishing PWID population is consistent with our findings. In the < 25 years old prisoners only 5.9% (12/203) reported ever using heroin and only 0.5% (1/203) reported injecting heroin compared to 39.7% (25/63) and 25.4% (16/63) in participants aged 45–49 years. Also in the study by Christensen et al., the mean age of the PWID was 28 years [20] whereas in our study it was 41 years. In fact, some of the older prisoners had also participated in the previous study.

A decline in the number of active PWID has also been observed in other countries that are comparable to Denmark. In Norway the number of active PWID decreased from around 10,000 in 2002 to 8,500 in 2013 [31] and in the Amsterdam Cohort Studies a low level of inflow of new PWID has seen the number of active PWID decline since 1990 to where only around 20% of the cohort were actively injecting in 2010 [32].

Another possible explanation for the declining number of PWID in Danish prisons over the last 20 years could be the improvements that have made been in OST coverage during that period. Heroin users have a high level of, especially income-generating, criminal activity [33, 34] and being on OST has been shown to reduce criminal activity in this group [35–37]. In Denmark the number of drug users on OST rose from less than 1,000 in 1985 to 7,400 in 2011[38] where it stabilized[30] and the same trend is seen when comparing the data in our study with the study by Christensen et al. Over the 20 years between the two studies the fraction of PWID who had ever received OST rose from 12.6% to 85.3% [20].

The low overall incidence of HCV infection in our study is in line with a recent study from Scotland in which the authors tested inmates in 14 high security prisons for HCV using the same methods as in our study and found an incidence of 0.6–0.9% [39]. The low incidence rate was consistent with the low number of inmates who reported injecting while in prison and according to the prison staffs finding needles in the prisons had become a very rare occurrence. However, the incidence rate of 18.1–24.5/100 PY among antibody-negative PWID was high compared to the Scottish study by Taylor et al, who found an incidence rate of 3.0–4.3/100 PY among antibody-negative PWID [39], but it was comparable to the incidence rates of 25.2% among PWID found by Christensen et al 20 years ago [20]. This would suggest that the risk behavior among incarcerated PWID in Denmark is still high, despite a high degree of OST coverage, indicating a need for improved preventive measures in Danish prisons to reduce HCV transmission among PWIDs. This could be access to clean injection equipment in prison, or perhaps more feasible opt out test for HCV at admission and prompt in prison treatment to reduce the HCV infection pool in prison.

Injection drug use as a primary risk factor for HCV infection in the prison population is in concordance with most studies [21] and given that the majority of HCV infected prisoners were either not diagnosed or linked to care, introduction of formalized screening on the basis of intravenous drug use should be instituted in Danish prisons. The association between HCV infection in prisoners and length of imprisonment has been shown in other studies [20, 40]. The high incidence rate over the last 20 years among PWID in Danish prisons, based on our study and the study by Christensen et al., would suggest that incarceration is still a period of high transmission risk. In conclusion, this study showed a significant decline in prevalence and incidence of HCV infection among Danish prison inmates compared to 20 years ago. Most of the HCV infections were known and had been diagnosed prior to the study, but less than half were attending care. The low number of cases is due to a low prevalence of PWID in Danish prisons, which most likely reflects a both declining and ageing population of PWID in the Danish prisons and in the general Danish population and a high degree of OST coverage among Danish PWID—also in prison. Risk factors for HCV infection were injection drug use, older age and longer lifetime duration of imprisonment.

## Supporting information

S1 File Questionnaire. (PDF)
