# Supplementary material for: Incidence, prevalence and risk factors for hepatitis C in Danish prisons
Source: PLoS One. 2019 Jul 26;14(7):e0220297. doi: 10.1371/journal.pone.0220297 (PMC6660074; doi:10.1371/journal.pone.0220297)
Supplement: S1 File — (PDF) [file pone.0220297.s001.pdf]

# Questionnaire

DATE_______ Prison_______Completed by_______ CASENR__________
INITIALS _______ Born in DK __ Yes ___No If no: country of birth __________
Age (years) _______
Weight (kg) _______ Height (cm) _______ Waist (cm) _______
Date of current imprisonment____/____/_______ Scheduled release from prison ____/____/_______
(Date/Month/Year) (Date/Month/Year)
Total number of years in prison_______

**1.Infection**
Have you ever been tested for hepatitis ___ Yes ___ No ___ Don't know If yes: year of last test ________
Are you infected with hepatitis C ___ Yes ___ No ___ Don't know If yes: year of diagnosis _______
Are you infected with hepatitis B ___ Yes ___ No ___ Don't know If yes: year of diagnosis _______
Have you ever been tested for HIV ___ Yes ___ No ___ Don't know If yes: year of last test ________
Are you infected with HIV ___ Yes ___ No ___ Don't know If yes: year of diagnosis _______

If infected, are you retained in care ___ Yes ___ No ___ Don't know If yes, for ___ Hepatitis C / ___ hepatitis B / ___ HIV

Have you been vaccinated against hepatitis ___ Yes ___ No ___ Don't know
If yes, have you been vaccinated against ___ Hepatitis A ___ Hepatitis B _______ Year of last vaccination?

**2.Drug use**
Have you ever used anabolic steroids or other performance enhancing drugs? ___ Yes ___ No If yes, have you ever injected anabolic steroid or other performance enhancing drugs? ___ Yes ___ No If yes, have you injected steroids or other performance enhancing drugs in prison? ___ Yes ___No
Have you ever smoked hashish on a daily basis? ___ Yes ___ No If yes, how many grams per week ___ For how many years ___
Have you ever used illicit drugs (other than hashish)? ___ Yes ___ No (if no go to **3.**)
What drugs have you used? ___ Heroin ___ Cocaine ___ Amphetamine ___ other: _______________________
Have you ever injected drugs? ___ Yes ___ No
Have you ever shared needles? ___ Yes ___ No If yes, in prison? ___ Yes ___ No
Have you ever shared spoons? ___ Yes ___ No If yes, in prison? ___ Yes ___ No
Have you ever shared cottons? ___ Yes ___ No If yes, in prison? ___ Yes ___ No
Have you ever shared water? ___ Yes ___ No If yes, in prison? ___ Yes ___ No
Have you taken an overdose within the last year? ___ Yes ___ No
Have you ever had an infection after injecting drugs? ___ Yes ___ No
Have you ever been on opioid substitution therapy? ___ Yes ___ No
If yes: Methadone years ___ ml ___ Subutex/suboxone years ___ mg ___
Have you been on OST before the current incarceration? ___ Yes ___ No Are you currently on OST ___ Yes ___ No

**3. Other**
Have you ever had a tattoo? ___ Yes ___ No If yes, how many? ___ Done by an amateur? ___ Yes ___ No While in prison ___ Yes ___ No
Have you ever consumed more than 5 units of alcohol daily? ___ Yes ___ No If yes, for how many years? ___ What year did you stop drinking? ____
What is your sexual orientation? ___ Heterosexual ___ Bisexual ___ Homosexual
Have you ever been with a prostitute? ___ Yes ___ No ___ Don't know
Have you ever worked as a prostitute (women)? ___ Yes ___ No ___ Don't know
Have you ever had a sexually transmitted disease? ___ Yes ___ No ___ Don't know
If yes: Gonorrhea ___ Yes ___ No Syphilis ___ Yes ___ No Genital herpes ___ Yes ___ No Chlamydia ___ Yes ___ No Condyloma ___ Yes ___ No
Do you suffer from: Diabetes ___ Yes ___ No Hypertension ___ Yes ___ No Cardiac disease ___ Yes ___ No

Do you suffer from any other diseases? ______________________________________________________________

Do you receive any medication? ______________________________________________________________

DATE AND SIGNITURE: __________________________
